# Sexual and Gender-Diverse Individuals Face More Health Challenges during COVID-19: A Large-Scale Social Media Analysis with Natural Language Processing

**DOI:** 10.34133/hds.0127

**Published:** 2024-09-06

**Authors:** Zhiyun Zhang, Yining Hua, Peilin Zhou, Shixu Lin, Minghui Li, Yujie Zhang, Li Zhou, Yanhui Liao, Jie Yang

**Affiliations:** ^1^ Department of Big Data in Health Science School of Public Health, Zhejiang University School of Medicine, Hangzhou, China.; ^2^ Department of Epidemiology, Harvard T.H. Chan School of Public Health, Boston, MA, USA.; ^3^ Department of Biomedical Informatics, Harvard Medical School, Boston, MA, USA.; ^4^ Division of General Internal Medicine and Primary Care, Department of Medicine, Brigham and Women’s Hospital, Boston, MA, USA.; ^5^ Thrust of Data Science and Analytics, The Hong Kong University of Science and Technology (Guangzhou), Guangzhou, China.; ^6^ Department of Psychiatry, Sir Run Run Shaw Hospital, Zhejiang University School of Medicine, Hangzhou, China.

## Abstract

**Background:** The COVID-19 pandemic has caused a disproportionate impact on the sexual and gender-diverse (SGD) community. Compared with non-SGD populations, their social relations and health status are more vulnerable, whereas public health data regarding SGD are scarce. **Methods:** To analyze the concerns and health status of SGD individuals, this cohort study leveraged 471,371,477 tweets from 251,455 SGD and 22,644,411 non-SGD users, spanning from 2020 February 1 to 2022 April 30. The outcome measures comprised the distribution and dynamics of COVID-related topics, attitudes toward vaccines, and the prevalence of symptoms. **Results:** Topic analysis revealed that SGD users engaged more frequently in discussions related to “friends and family” (20.5% vs. 13.1%, *P* < 0.001) and “wear masks” (10.1% vs. 8.3%, *P* < 0.001) compared to non-SGD users. Additionally, SGD users exhibited a marked higher proportion of positive sentiment in tweets about vaccines, including Moderna, Pfizer, AstraZeneca, and Johnson & Johnson. Among 102,464 users who self-reported COVID-19 diagnoses, SGD users disclosed significantly higher frequencies of mentioning 61 out of 69 COVID-related symptoms than non-SGD users, encompassing both physical and mental health challenges. **Conclusion:** The results provide insights into an understanding of the unique needs and experiences of the SGD community during the pandemic, emphasizing the value of social media data in epidemiological and public health research.

## Introduction

The COVID-19 pandemic has posed tremendous pressure on global health systems, leading to issues such as resource constraints and overcrowding in medical facilities [1]. These challenges are particularly acute for vulnerable communities, including sexual and gender-diverse (SGD) individuals, who frequently face systemic inequality [2]. Prior studies have reported that individuals from these communities encounter increased barriers to healthcare access due to both overt and systemic discrimination, as well as inadequate health insurance coverage [1,3]. They often have a higher prevalence of chronic conditions that are associated with severe COVID-19 outcomes, such as diabetes [4], cardiovascular diseases, and respiratory conditions like asthma [5,6]. In addition, systemic factors also placed these individuals in socially or emotionally challenging environments, heightening their risk for severe mental health issues [7].

This backdrop of heightened vulnerability underscores the crucial role that vaccine availability and acceptance play in curbing the spread of COVID-19 among SGD populations [8,9]. Existing research on vaccine hesitancy often overlooks or misrepresents these individuals [10], despite the fact that clinical and social factors contribute to their vaccine acceptance [11]. For instance, concerns over underlying health conditions [10] and considerations of vaccine efficacy and safety [12] all shape their attitudes toward vaccination. It is crucial, therefore, to target SGD individuals specifically, aiming to enhance their vaccine acceptance by deeply understanding their stance.

However, research on the health of SGD individuals during the COVID-19 pandemic faces a notable gap in both depth and breadth. Most studies rely on online surveys and questionnaires [13,14], constrained by the inherent biases of questionnaire design and the prolonged intervals of data collection. Moreover, research utilizing electronic health records typically focuses on specific symptoms of severe COVID-19, influenced by hospital admission rates and delays in gathering SGD data [15,16], thus lacking in generalizability. Additionally, the access to electronic health records is highly restricted, limiting the coverage of patients [17]. A major challenge in conducting comprehensive health status analysis for SGD populations lies in selecting large-scale, representative cohorts, which underscores the potential of alternative data sources, such as social media, which has been increasingly validated as a valuable tool in public health research [18–20]. Social media-related studies cover topics ranging from mental healthcare [21–23] to disease symptoms [24] and public acceptance of treatments [25–27], in the context of both COVID-19 and previous health crises such as H1N1 [28] and Zika [29]. Meanwhile, new deep learning-based language models, pipelines, and datasets [30–33] offer opportunities to analyze the massive textual information from social media platforms. This synergy between natural language processing (NLP) and social media analytics opens up novel avenues for research that span both data collection and analytical interpretation [18].

Within this context, our study leverages large-scale Twitter data and NLP methodologies to scrutinize the health and well-being of SGD individuals during the COVID-19 pandemic. We address 3 principal research questions: (a) What are the predominant topics discussed by SGD Twitter users during the pandemic? (b) How concerned are SGD individuals about pandemic precautions, such as mask-wearing and vaccination? (c) Do SGD individuals face more acute symptom risks and mental health challenges compared to non-SGD individuals during the pandemic?

To address the questions above, we employ Latent Dirichlet Allocation (LDA) models to discern public discussion themes and track their temporal evolution. Named Entity Recognition (NER) and Targeted Sentiment Analysis (TSA) models—both grounded in advanced NLP techniques and trained on Twitter-specific datasets—are used to compare vaccine perceptions between SGD and non-SGD individuals. We also identify and analyze Twitter users who have self-reported a COVID-19 diagnosis to compare health outcomes across SGD and non-SGD groups. Our preliminary results underline that SGD individuals manifest notably elevated symptomatology and mental health challenges, emphasizing an imperative for specialized interventions.

## Methods

### Experimental design

This cohort study collected a comprehensive dataset from February 2020 to April 2022, with the data collection process adhering to Twitter’s terms of service. Ethical approval was secured from the Institutional Review Board of Zhejiang University. An overview of data distribution and study design is provided in Fig. S1. SGD users were identified through user profiles, and topic modeling techniques were employed to analyze the content. Further statistical analyses were performed to understand their sentiments regarding COVID-19 vaccines, compare self-reported symptoms between SGD and non-SGD users, and investigate their mental health status.

### Data collection and selection

This study collected tweets through leveraging tweet IDs from a public coronavirus Twitter dataset [34], which follows specified accounts and collects real-time tweets mentioning specific keywords. We instituted a filtering process where tweets containing URLs were excluded to attenuate the impact of news and automated bot activities. Subsequently, we focused on identifying tweets from SGD users, including lesbian, gay, bisexual, transgender, queer, intersex, and asexual individuals [35]. SGD users were filtered through user profiles using keyword filtering and regular expression matching (Table S1): (a) User profiles must contain SGD-related keywords. (b) There should be no negation words before or after the keywords. (c) The keywords should not be preceded or followed by terms such as “advocator” and “supporter” as some users may advocate for SGD rights without necessarily being SGD themselves. A manual validation process was conducted on a subset of 500 selected SGD users, achieving a classification accuracy of 93.8%. We also evaluated the baseline characteristics of geographic information on the validation subset (Table S2).

### Statistical analysis

All statistical tests were conducted using Python 3.8, and were 2-tailed tests, with significance levels adjusted using Bonferroni corrections for multiple comparisons.

#### Topic modeling

Given the imbalanced dataset with a disproportionate number of tweets from non-SGD users, we performed a random under-sampling to achieve parity in tweet numbers (*n* = 2,296,289 for both groups) and sensitivity analysis was applied to verify the stability of the under-sampling (Supplementary Methods and Table S4). The random sampled tweets were then preprocessed through (a) removing the mention symbol “@” and the quoted usernames, (b) removing stop and short words with less than 2 letters, (c) applying word lemmatization, (d) adding bigrams and trigrams that co-occur more than 5 times, and (e) removing short tweets containing less than 5 tokens.

After preprocessing, 3,498,468 tweets were subjected to LDA [36] using the Gensim package [37]. Model selection criteria included both topic coherence and model perplexity, tested over a range of 10 to 50 topics. The topic number was set to 12 in our case according to experiments on balancing coherence and perplexity scores (Fig. S2). Topic validity was further confirmed through visualization using pyLDAvis [38] and manual inspection of the top 20 keywords per topic (Table S3). To compare the discussion differences between SGD users and non-SGD users on a specific topic, we applied *Scattertext* [39] to visualize the word frequency.

#### Sentiment analysis of vaccines

We used a pre-trained language model, COVID-Twitter-BERT [30], which was a BERT-LARGE structure pre-trained on 160 million COVID-19-related tweets, as the backbone for our named entity analysis (NER) and TSA models. A linear layer and a SoftMax function were added to the end of CT-BERT [40] to predict the span of each vaccine entity for the NER model. The encoder of BERT-SPC [41] was replaced with CT-BERT for the TSA model. The models were fine-tuned on the training set of the Medical Entities and Targeted Sentiments on COVID-19-related tweets (METS-CoV) dataset [32] using NCRF++ [42]; both are part of our prior work. This dataset included annotations for vaccine entities and their corresponding sentiment labels in tweets. The performance of the NER and TSA models was tested on the vaccine entity from the METS-CoV test set and resulted in an F1 score of 90.44% and an accuracy of 79.15%, respectively. As most of the recognized vaccine entities were informal expressions or misspelled, we manually incorporated the expressions of vaccine entities (details provided in Table S5) and selected the 4 most frequently mentioned COVID-19 vaccines (Moderna, Pfizer, AstraZeneca, and Johnson & Johnson) for in-depth analysis.

#### Symptom extraction and identification

Tweets that self-reported COVID-19 diagnoses were identified using lexicon filtering (details provided in Table S6). Tweets not written in the first person or contained negative or uncertain expressions (e.g., wonder, thought, might, etc.) before and after the keywords were filtered out through regular matching. For each selected user, we determined the date of diagnosis based on the content of the first self-report tweet. If the date of diagnosis was not specified in that tweet, we assumed that the time of tweeting was the time of diagnosis.

Tweets posted before and after 30 days of their self-report date were collected for users who self-reported COVID-19 diagnoses. We then screened these tweets using a COVID-19 symptom lexicon developed by Wu et al. [43], which contains commonly used synonyms and colloquial variants on social media that pertain to symptoms and their associated affected organs or systems. For the identification of mental health-related tweets, we utilized an exhaustive mental health lexicon [44], which has been rigorously validated by professionals in the fields of psychiatry and psychology. This lexicon encompasses 231 keywords distributed across 4 major mental health conditions: anxiety, depression, insomnia, and substance use disorders.

## Results

We downloaded a total of 471,371,477 tweets from the public COVID-19 Twitter dataset. After excluding tweets with URLs, the dataset was narrowed down to 169,669,346 tweets. Within this set, 2,296,289 tweets originated from 251,455 SGD users and 167,373,057 tweets originated from 22,644,411 non-SGD users (details provided in Table S1).

### Topic distribution and discrepancies

**Fig. 1.**
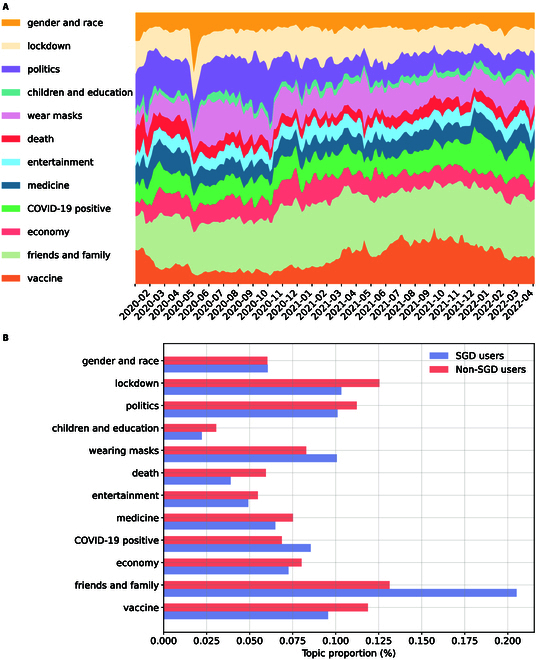
Topic distributions in COVID-19-related tweets. (A) The topic proportion distribution of SGD user-posted tweets over time. (B) Topic distributions of tweets posted by SGD users and non-SGD users.

After preprocessing, 3,498,468 tweets were subjected to the topic model, and the top 5 most discussed topics were “friends and family” (16.8%, 95% CI, 16.7% to 16.9%), “lockdown” (11.4%, 95% CI, 11.3% to 11.5%), “vaccine” (10.7%, 95% CI, 10.6% to 10.8%), “politics” (10.7%, 95% CI, 10.6% to 10.8%), and “wearing masks” (9.2%, 95% CI, 9.1% to 9.3%). Notably, these trends varied over time and exhibited specific peaks, as smoothed by a 7-day moving average (Fig. 1A). Besides, topic fluctuations reflect big events during the pandemic. For instance, the sharp increase in discussions on “gender and race” in May 2020 corresponds to heightened concerns about racial equity [45] following the police killing of George Floyd, an unarmed Black civilian [46]. Similarly, the U.S. presidential election and subsequent debates over COVID-19 policies [47] caused a spike in the topic “politics” in November 2020.

When comparing topic frequencies between SGD and non-SGD users using *χ*^2^ test with Bonferroni-adjusted significance level *P* < 0.004, the data indicate that discussions about “friends and family” were significantly more prevalent among the former (20.5% vs. 13.1%, *P* < 0.001) (Fig. 1B). For a deeper insight into the discussion variations, we used *Scattertext* [39] to visualize the word frequencies (Fig. S3). The result highlights that terms regarding family members occur frequently, with SGD users often mentioning “partner” while non-SGD users more frequently use terms like “daughter” and “baby”. Furthermore, SGD users express a range of emotions more frequently, especially negative ones like “anxiety”, “upset”, “angry”, and “depression”. For other topics, SGD users were more likely to talk about “wear masks” (10.1% vs. 8.3%, *P* < 0.001) and “COVID-19 positive” (8.6% vs. 6.9%, *P* < 0.001), while non-SGD users discussed other topics like “vaccine” (9.6% vs. 11.9%, *P* < 0.001) and “lockdown” (10.3% vs. 12.5%, *P* < 0.001) more often.

### Attitude toward COVID-19 vaccines

Table 1 shows the distribution of 3 category sentiments (positive, neutral, and negative), wherein the majority were characterized as neutral. Notably, sentiments toward the Pfizer vaccine exhibited the highest frequency of positive evaluations. Contrarily, attitudes toward the AstraZeneca vaccine appeared the most polarized among SGD users when contrasted with non-SGD users. Utilizing an independent samples *t* test for statistical analysis, we found that SGD users displayed significantly higher proportions of positive sentiments for all 4 vaccine types including Moderna (tweets, *n* [%]: 610 [12.6] vs. 28,828 [8.6], *P* < 0.001), Pfizer (tweets, *n* [%]: 984 [12.9] vs. 58,109 [8.8], *P* < 0.001), AstraZeneca (tweets, *n* [%]: 158 [11.0] vs. 16,227 [3.8], *P* < 0.001), and Johnson & Johnson (tweets, *n* [%]: 208 [9.3] vs. 11,482 [6.5], *P* < 0.001). Furthermore, the proportions of negative sentiments for both the Moderna (tweets, *n* [%]: 182 [3.7] vs. 21,271 [6.4], *P* < 0.001) and Pfizer (tweets, *n* [%]: 327 [4.3] vs. 51,669 [7.8], *P* < 0.001) vaccines were significantly lower among SGD users compared to the non-SGD group.

**Table 1.** Percentage of positive and negative sentiments in vaccine-related tweets

Vaccine	Positive			Negative		
	Tweets, *n* (%) [95% CI^a^]		*P* value^b^ (SGD greater)	Tweets, *n* (%) [95% CI]		*P* value (SGD smaller)
	SGD	Non-SGD		SGD	Non-SGD	
**Moderna**	610 (12.6) [11.6–13.5]	28,828 (8.6) [8.5–8.7]	<0.001	182 (3.7) [3.2–4.3]	21,271 (6.4) [6.3–6.5]	<0.001
**Pfizer**	984 (12.9) [12.2–13.7]	58,109 (8.8) [8.7–8.8]	<0.001	327 (4.3) [3.8–4.8]	51,669 (7.8) [7.7–7.9]	<0.001
**AstraZeneca**	158 (11.0) [9.4–12.7]	16,227 (3.8) [3.8–3.9]	<0.001	68 (4.8) [3.7–5.9]	11,071 (2.6) [2.6–2.7]	0.999
**Johnson & Johnson**	208 (9.3) [8.1–10.5]	11,482 (6.5) [6.4–6.6]	<0.001	127 (5.6) [4.7–6.6]	11,191 (6.3) [6.2–6.4]	0.101

^a^ We used the ratio *t* test to calculate 95% confidence intervals and the independent samples *t* test to calculate significance.

^b^ Significance was set at *P* < 0.006 after Bonferroni correction.

### Physical and mental health status

We identified 2,098 SGD and 100,366 non-SGD users who self-reported COVID-19 diagnoses (the overview of users’ filtering process is provided in Fig. S4). Analysis of tweets within a 30-day window surrounding the self-reported date yielded mentions of 69 unique symptoms, implicating 15 distinct organ systems or physiological functions (Supplementary File 1). An independent samples *t* test showed that the frequency of mentions for 61 of these 69 symptoms was significantly higher (*P* < 7.25×10^−4^, Bonferroni adjusted) among the SGD cohort compared to the non-SGD group.

**Fig. 2.**
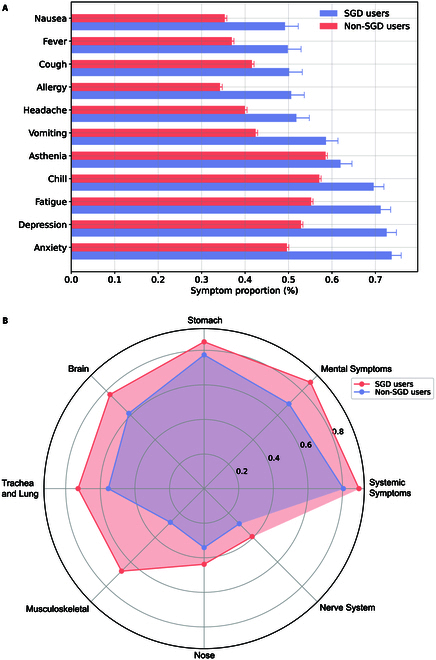
Symptom distributions in users who self-report COVID-19 positive. (A) Symptom distributions of SGD and non-SGD Twitter users. (B) Distribution of the 8 most affected organs or systems of SGD and non-SGD Twitter users.

We then calculated mention rates for each symptom in both groups. Figure 2A displays mention rates for symptoms cited by more than 1,000 individuals in the SGD group and 35,000 in the non-SGD group. Symptoms most frequently mentioned—such as anxiety, nausea, and allergic reactions—had higher prevalence among SGD individuals. Figure 2B further shows that mention rates for symptoms related to mental and musculoskeletal health were especially elevated in the SGD individuals, followed by mental symptoms, trachea and lung, and brain.

For mental health analysis, from 137,860 mental health-related tweets contributed by SGD individuals and 7,647,024 tweets contributed by non-SGD individuals, we identified 1,984,317 tweets related to anxiety, 5,258,324 related to depression, 586,648 related to insomnia, and 200,905 related to substance use disorders.

**Fig. 3.**
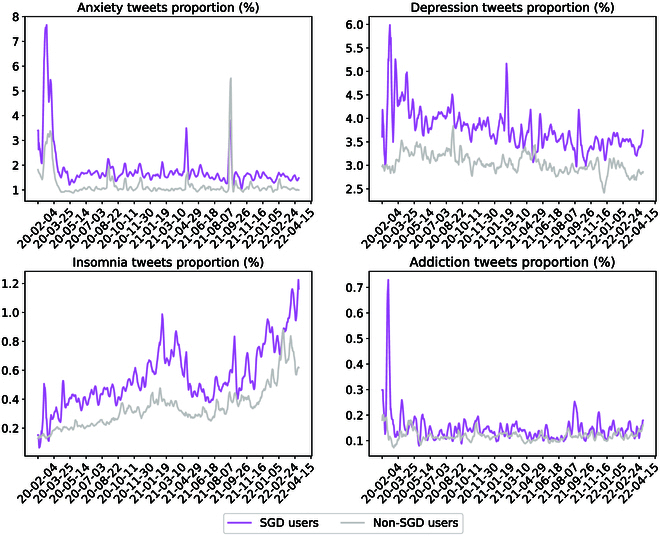
The proportion of tweets concerning mental health to the total number of tweets. We used a 7-day moving average to smooth the curve.

Figure 3 shows the temporal distribution of these mental health-related tweets. We observed an initial surge in tweets concerning anxiety, depression, and substance use at the onset of the pandemic, followed by a stabilization to baseline levels. Contrastingly, the proportion of tweets pertaining to insomnia demonstrated a continual increase over time. Given that the tweet distribution over time deviated from normality, we employed 2-tailed Wilcoxon matched-pairs signed-ranks tests (Table 2), and it turned out that SGD users exhibited higher prevalence of anxiety (% tweets, median [IQR]: 1.58 [0.53] vs. 1.05 [0.16], *P* < 0.001), depression (% tweets, median [IQR]: 3.63 [0.89] vs. 3.02 [0.31], *P* < 0.001), insomnia (% tweets, median [IQR]: 0.52 [0.33] vs. 0.32 [0.15], *P* < 0.001), and addiction (% tweets, median [IQR]: 0.13 [0.10] vs. 0.12 [0.02], *P* < 0.001) symptoms.

**Table 2.** The daily proportion of mental health-related tweets of SGD and non-SGD users. *W* represents the sum of the ranks of the differences above zero.

Mental symptoms	SGD (% tweets), median (IQR^a^)	Non-SGD (% tweets), median (IQR)	*W*	*P* value^b^
**Anxiety**	1.585 (0.530)	1.047 (0.158)	317,574	<0.001
**Depression**	3.634 (0.885)	3.024 (0.315)	302,044	<0.001
**Insomnia**	0.523 (0.325)	0.320 (0.154)	312,525	<0.001
**Addiction**	0.127 (0.103)	0.115 (0.024)	203,679	<0.001

^a^ We used the IQR and Wilcoxon matched-pairs signed-ranks test in the statistical analysis.

^b^ Significance was set at *P* < 0.016 after Bonferroni correction.

## Discussion

In this pioneering social media-based retrospective cohort study, we examined the differential impact of the COVID-19 pandemic on SGD individuals by analyzing a large corpus of pandemic-related tweets over 2 ^1^/_2_ years. Our methodological approach encompasses (a) the use of topic modeling to delineate concerns unique to SGD individuals; (b) the application of advanced deep learning-based NLP algorithms for sentiment analysis toward vaccines; and (c) a comparison of self-reported COVID-19 symptoms between SGD and non-SGD individuals. Through these avenues, our research aims to elucidate the unique challenges confronting SGD individuals during the pandemic and to inform targeted interventions designed to alleviate their physical and psychological burdens, and therefore enhance their well-being.

Our topic modeling results divulge a heightened focus among SGD individuals on themes like “friends and family”, where they tend to express negative emotions more frequently. Research indicates that pandemic policies, such as school closures and lockdowns, have severed some social connections, leaving SGD individuals more dependent on family members [3]. However, older individuals are often isolated as they are 4 times less likely to have children and SGD youth are forced to be at home with unsupportive parents [7]. Emotional and mental health harms may arise from the lack of supportive surroundings. We also observed that SGD individuals are more likely to discuss topics related to preventative health measures such as “mask-wearing” and “COVID-19 testing”. These observations align with previous research by Sears et al. [48] conducted in the United States. They found that SGD individuals prefer to wear masks (94.0% vs. 89.9%) and take COVID-19 tests (38.3% vs. 29.0%).

Our sentiment analysis further shows more positive attitudes toward vaccines within SGD individuals compared to their non-SGD counterparts, which is positively correlated with stronger vaccination stance [49,50]. This higher rate of vaccine adoption is corroborated by telephonic surveys and suggests greater compliance with public health directives within these communities [51,52]. These results suggest that SGD individuals exhibit a greater awareness of the importance of precautionary measures and prefer to comply with public health orders during the pandemic [53]. The higher willingness of SGD individuals to vaccinate may be linked to their altruistic tendencies [54] and higher levels of perceived health vulnerability [55]. Nonetheless, vaccination rates for SGD populations vary widely over different regions and ethnicities [10]. More efforts are needed to assess vaccination rates in these areas and improve the coverage for SGD individuals without insurance or documents.

In examining self-reported symptoms among COVID-19-positive individuals, our data reveal concordance with clinical studies [56–58] regarding the most frequently mentioned symptoms. However, we found a higher frequency of certain mild symptoms such as musculoskeletal and mental health issues within the SGD population, as compared to electronic health record-based studies [59]. This underscores the utility of social media as a complementary data source for capturing a broader spectrum of patient experiences that might not be adequately recorded in clinical settings. In addition, we noted that SGD individuals are more likely to experience more severe symptoms after COVID-19 infection. This may be due to inadequate health insurance coverage and higher-than-average rates of underlying diseases such as diabetes, and asthma, which can increase the risk of severe symptoms [5,6]. Besides, SGD users mentioned musculoskeletal symptoms (body pain, myalgia pain, arthralgia pain, etc.) at a particularly higher rate compared to non-SGD users. These symptoms are often associated with severe disease as they can be triggered by increased inflammatory factors (e.g., interleukin-6) during infection [60,61].

The psychological ramifications within SGD communities warrant nuanced attention, as our study indicates elevated rates of mental health symptoms than non-SGD groups during the pandemic, which is consistent with pre-existing literature employing the PHQ4 scale and online surveys [13,14,62]. In terms of temporal variations, the frequency of insomnia-related tweets exhibited a correlation with diagnosed COVID-19 cases, peaking in January 2021 and rising steadily from January through April 2022. These trends are congruent with clinical literature suggesting a high correlation between insomnia and COVID-19 infection [63–65]. Conversely, fluctuations in tweets related to other mental health conditions—namely, depression, anxiety, and addiction—appeared to be more significantly influenced by social determinants. For instance, a sudden spike in anxiety-related tweets occurred in September 2021, and the majority of the discussion was focused on the increase in fuel prices. This phenomenon has also been observed by previous research [44]. During the pandemic’s initial outbreak in February 2020, SGD communities experienced more pronounced spikes in symptoms of depression, anxiety, and addiction compared to their non-SGD counterparts. These exacerbated symptoms may be attributed to distinct and more severe social challenges confronting SGD individuals, such as limited access to supplies and healthcare [66]. Moreover, it is crucial to highlight the constrained social support networks often associated with SGD communities, which include family, partners, and peers. Such networks frequently lack the resilience and social capital to act as effective buffers against the immediate repercussions of both social changes and health crises [67].

We acknowledge several limitations in our study. First, the age and geographical distributions of Twitter users are skewed, introducing potential selection bias that may limit the external validity of our findings. For instance, individuals with lower socio-economic status or those of advanced age may be underrepresented on Twitter, thereby introducing a bias toward certain demographic groups [68]. Second, despite the application of advanced NLP models employing deep learning, our pipeline is susceptible to misclassification bias due to lexical ambiguity [69]. To assess this issue, we conducted a random selection of 500 tweets identified as originating from SGD individuals. Manual validation of these tweets suggests that 31 (6.2%) tweets may have been inaccurately categorized. Moreover, the composition of our non-SGD control group is subject to information bias; SGD users who have not publicly disclosed their identities on Twitter might be included, which could attenuate the observed effect sizes and affect the internal validity of our findings. Furthermore, our study is confined by the absence of pre-pandemic baseline data, largely due to Twitter’s data-sharing constraints. This results in a lack of temporal control, making it challenging to differentiate the health disparities between SGD and non-SGD groups directly attributable to the pandemic. Strict filtering criteria for users that self-reported COVID-19 positive may lead to a lower recall rate, resulting in selection bias among the remaining samples and an inability to represent the entire population. Moreover, the collected symptom descriptions may be subjective to the user and lack the strictness of evidence-based medicine, but they can serve as an auxiliary tool for public health analysis. These limitations should underscore the need for cautious interpretation.

In summary, this pioneering study employs various NLP techniques like NER, TSA, and LDA models, to provide an in-depth understanding of the experiences and health outcomes of SGD individuals during the COVID-19 pandemic. Our findings emphasize the importance of enhancing social and legal support for SGD individuals and informing public health interventions to address disparities during challenging times. The methodology and pipeline developed in this study can be applied to monitor the health of other populations, providing data-driven insights for more comprehensive public health services.

## Ethical Approval

This study was approved by the Institutional Review Board of the School of Public Health, Zhejiang University (ZGL202201-2).

## Data Availability

The codes used in this study can be accessed at https://github.com/zooay-zzy/COVID-twitter-SGD-analysis.
