# Supplementary material for: Sexual and Gender-Diverse Individuals Face More Health Challenges during COVID-19: A Large-Scale Social Media Analysis with Natural Language Processing
Source: Health Data Sci. 2024 Sep 6;4:0127. doi: 10.34133/hds.0127 (PMC11378377; doi:10.34133/hds.0127)
Supplement: Supplementary 1 — Methods Figs. S1 to S4 Tables S1 to S6 [file hds.0127.f1.zip › Supplementary Materials.docx]

**Methods**

**Sensitivity analysis of under-sampled non-SGD tweets in the topic model**. To assess the impact of different random seeds on topic distribution, we created two new samples of non-SGD tweets: Sample A, with the same number as the original sample (n = 2,296,289), and Sample B, with twice that number (n = 4,592,578). After preprocessing, 1,742,444 tweets remained in Sample A and 3,484,880 in Sample B. Then we evaluated the distribution of topics in these new corpora using the trained LDA model and compared it with the old distribution using chi square test (**Table S4**).


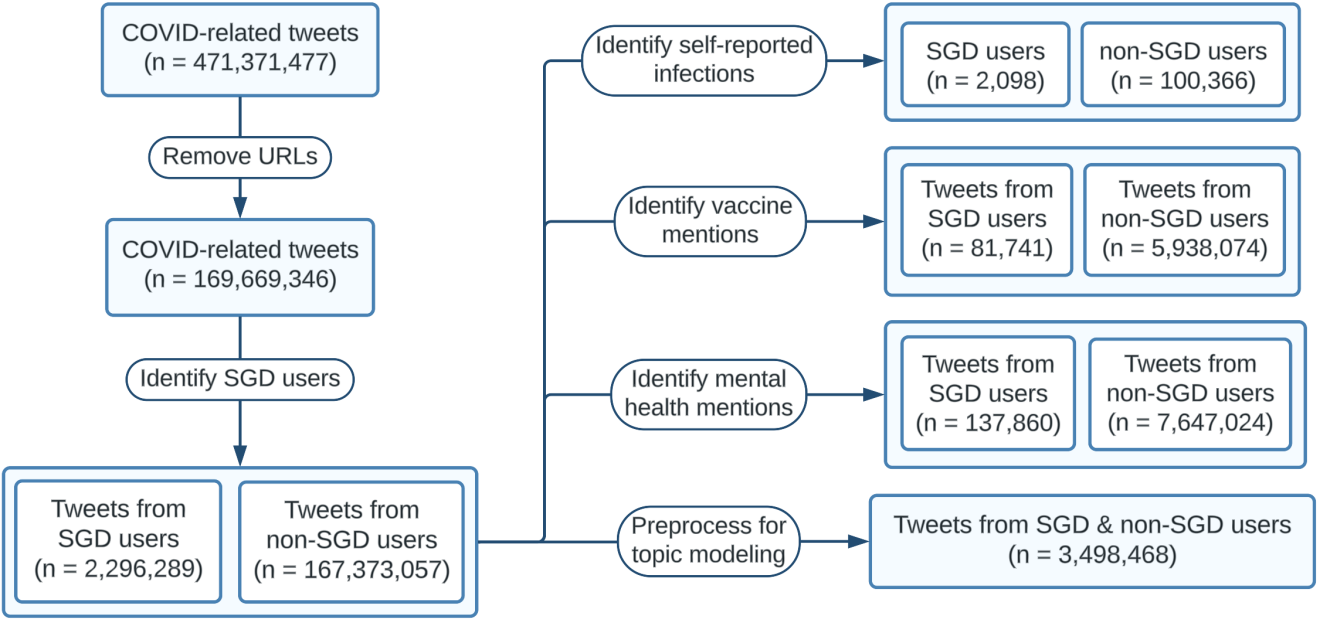


Figure S1. Data collection and distribution.


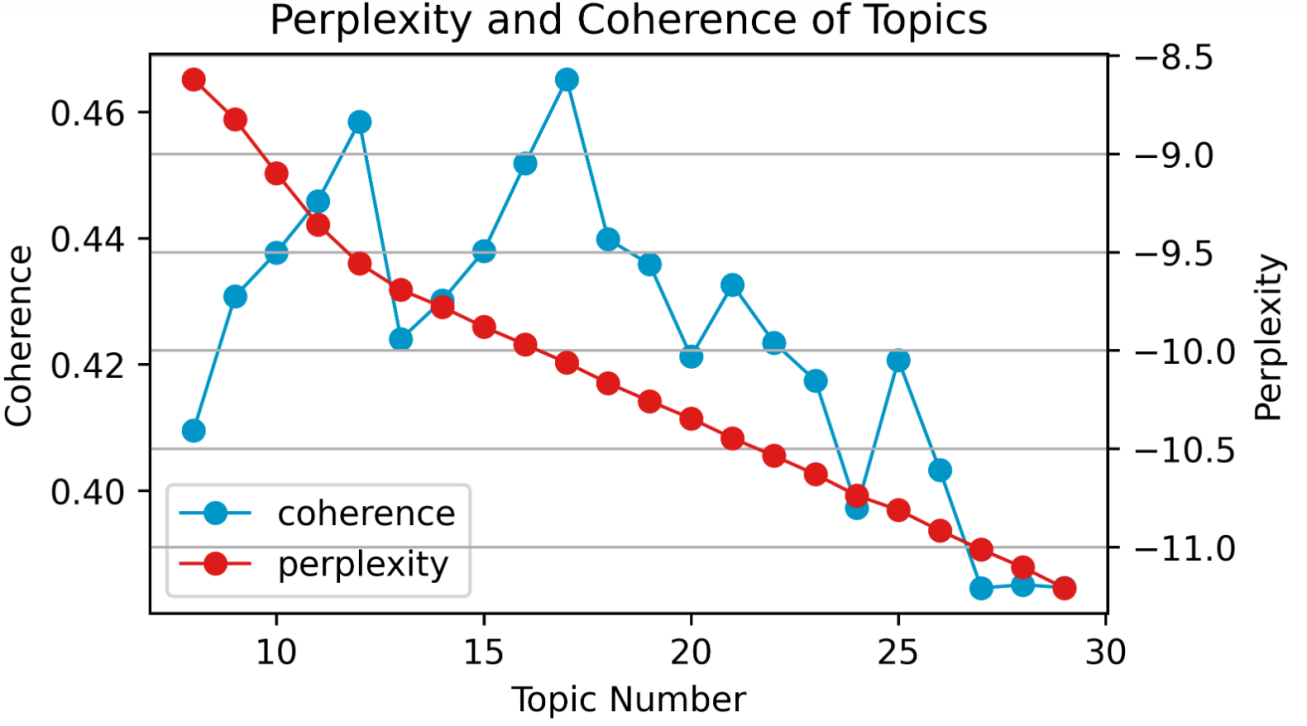


Figure S2. Perplexity and coherence score of different topics. Topic perplexity measures the generalizability of models to unseen data, and coherence measures the degree of semantic similarity between high-scoring words in topics.


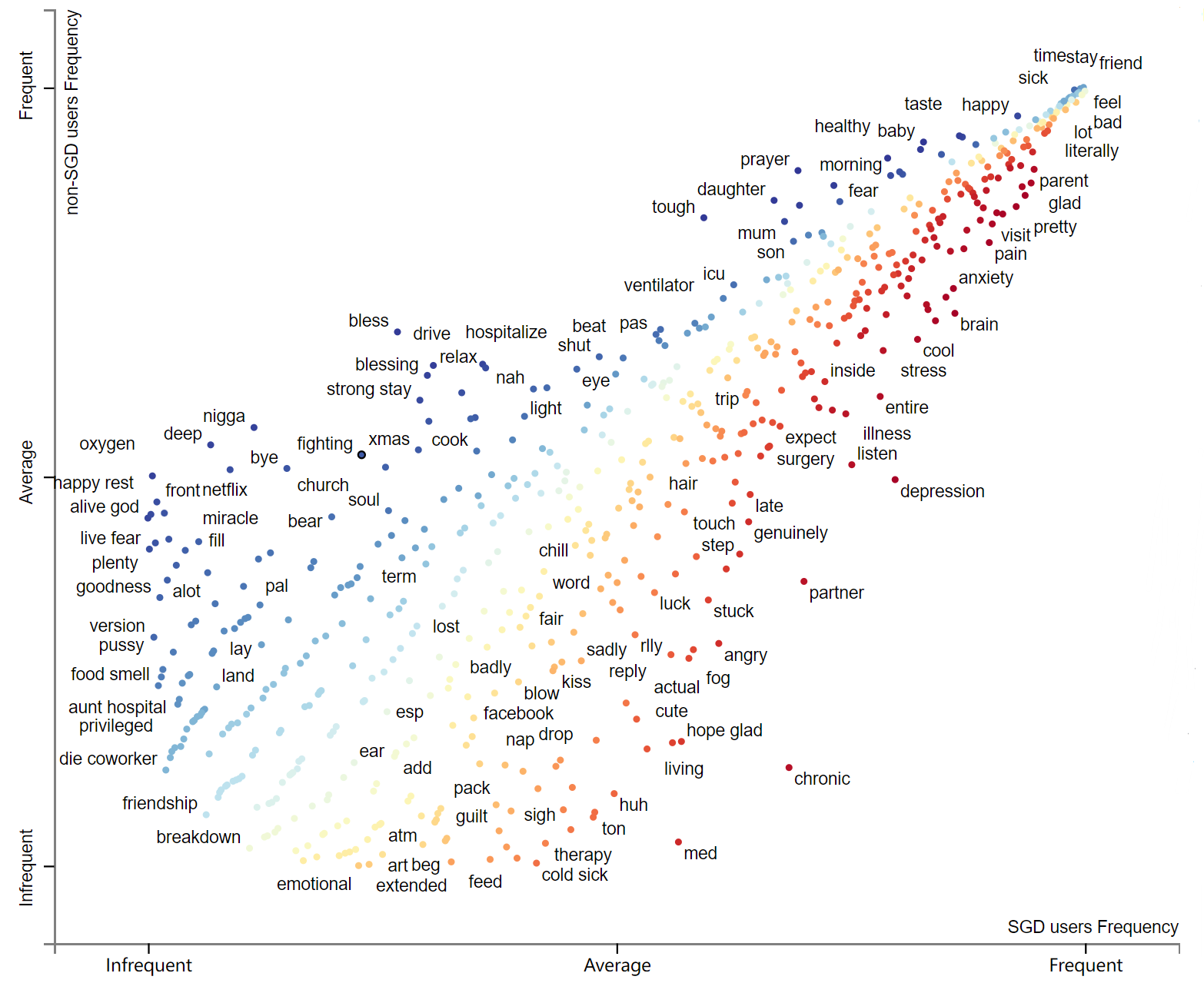


Figure S3. Visualization of word frequency in the topic “friend and family” using *Scattertext*. The x- and y- axes of terms are the dense ranks of their usage by SGD and non-SGD users respectively.


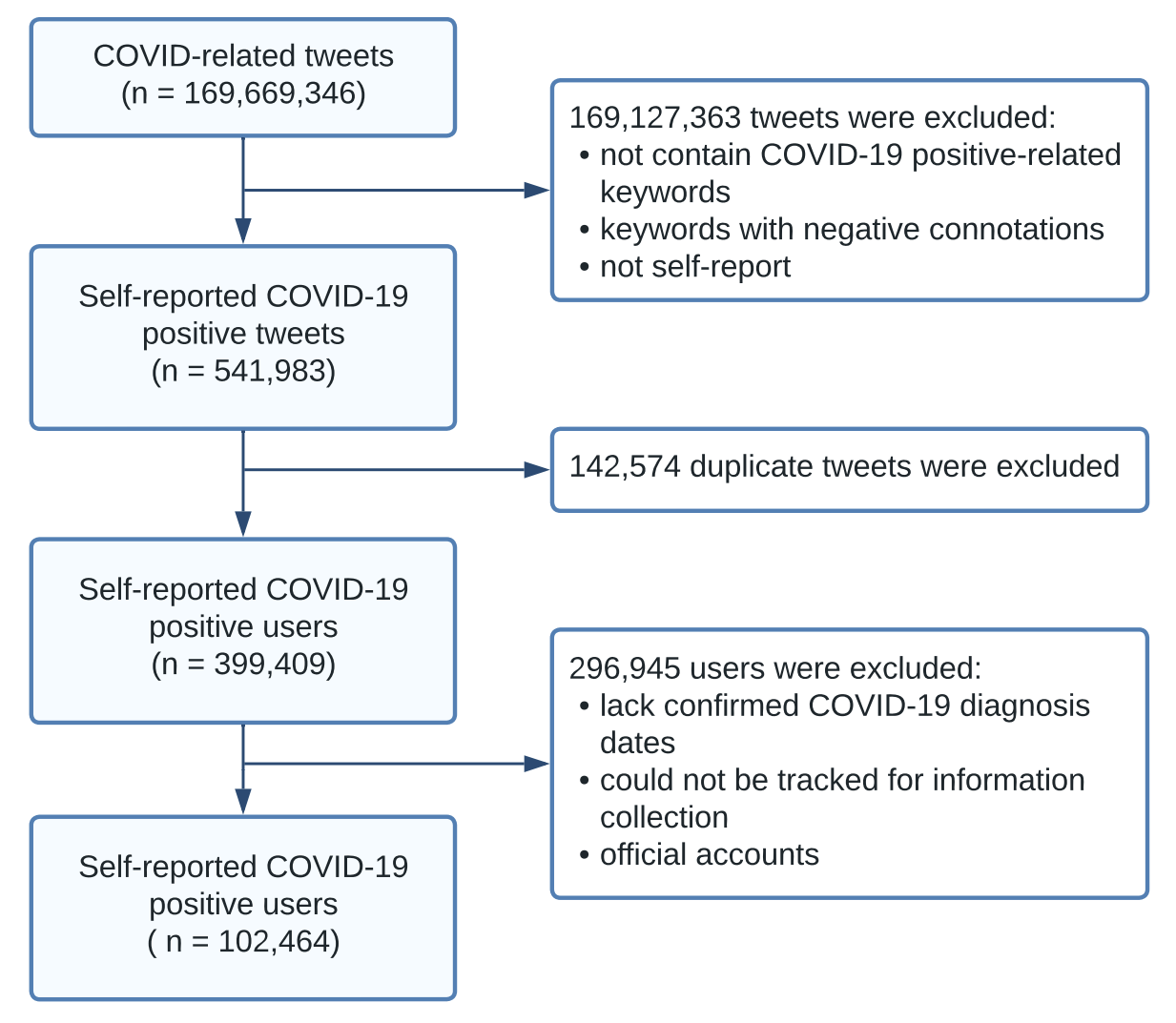


Figure S4. Self-reported COVID-19 positive users filtering process.

Table S1. Lexicon and rules for SGD selection. We used regular expressions to remove tweets with stop words before and after the SGD-related keywords to filter out negative expressions and SGD supporters but non-SGD individuals. "|" is used as a delimiter between lexicons.

| **Stop words in front** | **SGD lexicon** | **Stop words behind** |
| --- | --- | --- |
| not \| no \| none \| anti \| hate \| against \| pro \| advocate | lgbt \| lesbian \| gay \| bisexual \| transgender \| queer \| intersex \| asexual \| gender minority \| sex minority \| sex and gender minority | anti \| ally \| supporter \| advocator \| friendly |

Table S2. Geographic distribution of SGD and non-SGD users on the validation subset. The geographic information is extracted from the “place” field in tweet metadata but not the “location” field in “user” description, since the former is generated from GPS data.

|  | **SGD users (%) (n=500)** | **non-SGD users (%) (n=500)** | **P value^*^** |
| --- | --- | --- | --- |
| **Country** |  |  | 0.015 |
| United States | 295 (59.0) | 272 (54.4) |  |
| United Kingdom | 102 (20.4) | 98 (19.6) |  |
| Canada | 24 (4.8) | 22 (4.4) |  |
| Australia | 19 (3.8) | 18 (3.6) |  |
| India | 60 (12.0) | 90 (18.0) |  |

^*^P values were calculated using χ2 test.

Table S3. 12 topics given by the LDA model and associated top-20 keywords.

| **Topics** | **(value) Keywords** | **Example** |
| --- | --- | --- |
| vaccine | (0.081) vaccine, (0.022) vaccinate, (0.017) spread, (0.016) flu, (0.013) risk, (0.013) cdc, (0.012) vaccination, (0.012) die, (0.010) variant, (0.010) disease, (0.009) vaccinated, (0.009) prevent, (0.009) booster, (0.008) shoot, (0.008) shot, (0.007) catch, (0.007) science, (0.007) immune, (0.006) chance, (0.006) vax | “I see the occasional (or should that be odd?) posts by folk saying they will refuse a covid vaccine. terribly selfish. but fine as I am sure they will refuse treatment if they become ill. I do like to see darwinian natural selection in play.”  “It's proven that isn't true lol. you can catch covid, build antibodies, and still catch covid again because of variants. vaccines help build antibodies, thus creating stronger immunity, and when multiple people strengthen immunity through vaccination, herd immunity can happen.” |
| friends and family | (0.026) feel, (0.022) life, (0.020) friend, (0.020) time, (0.018) love, (0.016) family, (0.014) bad, (0.014) stay, (0.014) shit, (0.012) day, (0.011) live, (0.011) die, (0.010) hope, (0.010) lose, (0.008) mom, (0.008) lot, (0.008) start, (0.007) hard, (0.007) god, (0.007) month | “Please prioritize family sponsorships as in these hard times people need their loved ones around. being alone was never easy but hadn’t been so hard until these covid times.”  “I have no friends and havnt spoken to anyone in months coz I'd covid and I listen to your baking videos in the background to fill the void, thanks.” |
| economy | (0.033) job, (0.026) pay, (0.021) money, (0.015) bill, (0.014) relief, (0.010) business, (0.009) food, (0.009) lose, (0.008) cut, (0.008) company, (0.008) buy, (0.008) time, (0.007) tax, (0.007) check, (0.007) support, (0.006) cost, (0.006) struggle, (0.006) unemployment, (0.006) government, (0.005) month | “It doesn’t make sense to extend covid stimulus checks to those who are working; have not lost their jobs/businesses. the checks should target unemployed people who have lost their jobs; businesses to covid. state local govts must be given money to augment their lost revenue.”  “lol my landlord has been paid up to this month quit your free ride bs. my landlord has told me even if I got a job today it means nothing he is moving to evict. all because I was 25 dollars short 25 dollars short when the pandemic hit and lost my job. I got him their money later.” |
| covid positive | (0.073) test, (0.047) day, (0.031) week, (0.025) positive, (0.017) hour, (0.015) symptom, (0.013) time, (0.013) negative, (0.013) test_positive, (0.012) sick, (0.010) wait, (0.010) tomorrow, (0.008) appointment, (0.008) quarantine, (0.008) testing, (0.008) call, (0.007) leave, (0.007) house, (0.007) feel, (0.007) stay | “My brother got tested positive for covid almost two half weeks back, but remained asymptomatic. alhamdulillah. later my sister-in-law also tested positive with mild symptoms in quarantine at home. as a precaution, I'm going to get myself tested as well. hope it's a negative.”  “After possibly being exposed to covid + me having mild symptoms, we got tested for the first time today. results expected in 24-48 hours. hopefully we’re both negative” |
| medicine | (0.039) health, (0.027) care, (0.020) issue, (0.018) patient, (0.016) public, (0.016) hospital, (0.014) medical, (0.011) worker, (0.010) mental, (0.009) doctor, (0.009) nurse, (0.009) healthcare, (0.008) system, (0.008) staff, (0.008) community, (0.007) risk, (0.006) safety, (0.006) public_health, (0.006) service, (0.006) access | “You must work with nurses and other health care workers to make testing guidelines that ensure safety for frontline workers and patients. with regular testing now!”  “The pandemic isn’t over yet. all 50 states are seeing an increase in cases as we enter a potential fourth wave. hospital admissions have also climbed about 36% with deaths up 26%. vaccination efforts continue to be critical.” |
| entertainment | (0.020) play, (0.017) watch, (0.016) safe, (0.016) time, (0.015) game, (0.014) read, (0.013) stay, (0.012) book, (0.011) write, (0.010) hope, (0.010) video, (0.010) movie, (0.009) start, (0.008) team, (0.007) season, (0.007) event, (0.007) lot, (0.007) win, (0.007) forward, (0.006) release | “I'm used to reading multiple books (up to 10) at a time, and I know that sometimes I can't start a book/get into a book when I've got the others to finish, but that's not it w this one. and I'm not having trouble starting new books - if anything, this pandemic has made me read.” |
| death | (0.085) death, (0.030) die, (0.025) rate, (0.020) report, (0.019) news, (0.011) daily, (0.010) total, (0.010) infection, (0.010) record, (0.010) low, (0.009) disabled, (0.009) county, (0.009) gay, (0.008) day, (0.008) flu, (0.008) count, (0.008) panic, (0.008) attack, (0.008) population, (0.007) figure | “Take a look at death rates from flu, death rates before 2020, and covid death rates following infection rates. there is no question that increased death rates follow increased infection rates.”  “Me and bestie were gonna do the amazing race (no way that’s coming back thanks to covid) under the stipulation she did all the eating challenges. I’ll probably try in the future” |
| wear masks | (0.143) mask, (0.097) wear, (0.078) wear_mask, (0.034) social, (0.019) distancing, (0.017) social_distancing, (0.013) hand, (0.011) distance, (0.011) stay, (0.011) mandate, (0.008) store, (0.007) vaccinate, (0.007) public, (0.007) safe, (0.007) protect, (0.007) refuse, (0.006) social_distance, (0.005) wash, (0.005) guideline, (0.004) time | “I don’t know which year this picture is from, but I hope it’s 2019, since no one is wearing a mask or social distancing. we’re still waiting for a picture of you, pete, wearing a mask. be a good example.”  “I thought I could go on a camping trip but I can't go to any state that's remotely interesting because you idiots won't wear a mask.” |
| children and education | (0.074) school, (0.058) kid, (0.035) child, (0.021) student, (0.020) class, (0.017) parent, (0.016) online, (0.015) teacher, (0.010) college, (0.009) cancel, (0.008) teach, (0.008) asian, (0.007) south, (0.006) send, (0.005) rapid, (0.005) university, (0.005) attend, (0.005) excited, (0.005) board, (0.005) court | “Please cancel all state board exam and 12 class board exam ....please cancel it ....we are also student we can be also affected by corona.. why you didn't cancel all state board exam and 12 class board exam ...”  “No school for my grandchildren until they have been vaccinated against the coronavirus. home school is going to be the only way for now. ” |
| politics | (0.048) trump, (0.025) american, (0.019) vote, (0.018) lie, (0.017) biden, (0.015) die, (0.013) republican, (0.012) president, (0.011) kill, (0.010) dead, (0.010) election, (0.010) china, (0.009) call, (0.009) country, (0.008) america, (0.008) response, (0.007) gop, (0.007) blame, (0.007) care, (0.006) hoax | “Just a reminder under trump is out of control with no coordinated national response. over 141,000 americans.”  “Disappointed? this administration did nothing preemptive for this pandemic. governed by an old president, the health system on the hands of the very meaning of incompetence. What do we get? hmm” |
| lockdown | (0.084) lockdown, (0.015) time, (0.014) country, (0.012) travel, (0.011) start, (0.011) month, (0.010) restriction, (0.009) week, (0.009) government, (0.008) lock, (0.008) close, (0.008) rule, (0.007) happen, (0.007) live, (0.007) day, (0.006) plan, (0.006) city, (0.005) outbreak, (0.005) march, (0.005) summer | “England is no longer a free country. lord sumption described boris johnson's covid restrictions as the "most significant interference with personal freedom in the history of our country". Boris is a tyrant.”  “There's no point at all. that's what happened here in germany, we called it here light lockdown. only pubs, restaurants and cinemas were closed in november. and look where we are now. we've got 2 weeks straight now the highest infection and mortality rate in eu” |
| gender and race | (0.013) woman, (0.013) white, (0.012) life, (0.011) protest, (0.010) black, (0.009) police, (0.008) texas, (0.008) florida, (0.008) epidemic, (0.007) mental_health, (0.007) medium, (0.007) kill, (0.006) super, (0.006) anti, (0.006) spread, (0.006) war, (0.005) human, (0.005) crisis, (0.005) aid, (0.005) racism | “Yall, its 2020. we all really, really need to understand how gentrification works, and how gentrification is violent for black communities in atlanta. thanks to disaster capitalism, there is always a land grab post any major crisis and the same is poised to happen with covid.”  “there's a fucking pandemic going on, but sure blame the unions cause it's always about trashing teachers who are overwhelmingly women and seen as garbage.” |

Table S4. Topic distribution over different random samples.

|  | **Original sample (%) (n=1,742,385)** | **Sample A (%) (n=1,742,444)** | **Sample B (%) (n=3,484,880)** | **P value^*^** |
| --- | --- | --- | --- | --- |
| **Topics** |  |  |  | 0.081 |
| Vaccine | 206,950 (11.88) | 205,159 (11.77) | 412,512 (11.84) |  |
| Friends and family | 228,812 (13.13) | 229,475 (13.17) | 458,420 (13.15) |  |
| Economy | 139,718 (8.02) | 138,947 (7.97) | 278,209 (7.98) |  |
| COVID-19 positive | 119,737 (6.87) | 119,589 (6.86) | 239,316 (6.87) |  |
| Medicine | 131,090 (7.52) | 130,783 (7.51) | 261,970 (7.52) |  |
| Entertainment | 95,454 (5.48) | 95,876 (5.50) | 192,091 (5.51) |  |
| Death | 103,626 (5.95) | 103,769 (5.96) | 207,392 (5.95) |  |
| Wear masks | 144,446 (8.30) | 144,854 (8.31) | 289,289 (8.30) |  |
| Children and education | 53,268 (3.06) | 53,871 (3.10) | 107,920 (3.10) |  |
| Politics | 195,601 (11.23) | 195,544 (11.22) | 391,289 (11.23) |  |
| Lockdown | 218,585 (12.55) | 218,394 (12.53) | 436,592 (12.53) |  |
| Gender and race | 105,098 (6.03) | 106,183 (6.10) | 209,880 (6.02) |  |

^*^P values were calculated using χ2 test.

Table S5. Lexicon for vaccine incorporation.

| **Vaccine** | **Lexicon** |
| --- | --- |
| AZ | Astra Zeneca \| AstraZeneca \| Astrazeneca \| astrazeneca \| astra zeneca \| ASTRAZENECA \| astra Zeneca \| Astra Zenica \| AstraZenica \| Astra zenica \| astra zenica \| Astrazenica \| astrazenica \| astraderna \| Astra vaccination \| AstraZenna \| Astragenica \| AztraSeneca \| AztraZeneca \| Aztrazeneca \| AstroZeneca \| Astrozeneca \| AsatraZeneca \| Astra-Zeneca \| Astra-Zenica \| Astra-Zeneca vaccine \| astrozeneca \| aztrazeneca \| astazenica \| Astera Zeneca \| GenXZeneca shot \| Astra vaccine \| Astra jab \| AstraZ \| Astra Z \| AZ Vax \| AZ vax \| AZ vac \| AZ vaxxed \| AZ vaccine \| AZ vaccines \| AZ Vaccine \| AZ vaccination \| AZ vaccine shot \| AZ vaccinated \| AZ jab \| AZ jabs \| AZ Jabs \| AZ shot \| AZ shots \| AZ covid vaccine \| AZ Covid jab \| AZ Covid vaccine \| AZ COVID shot \| AZ COVID vax \| AZ COVID vaccines \| AZ COVID vaccine \| AZ covid jab \| AZ No jab \| AZ-Oxford vaccine \| AZ dose \| AZ vacc \| AZ one \| A-Z vaccine \| Az vaccine \| az vaccine \| Astra one \| AZN vaccine \| AZN COVID vaccine \| AZN COVID-19 Vaccine \| AZN COVID-19 vaccine \| AZN LN vaccine \| AZN shot \| AZN \| AZD1222 \| AZD1222 vaccine \| AZ/Oxford \| ChAdOx1 nCoV-19 vaccine \| ChAdOx1 nCoV \| ChAdOx1 \| Vaxzevria \| COVIDSHIELD \| COVISHIELD \| COVID shield \| CoviShield \| Covid Shield \| Covidshield \| Covishield \| Covid shield \| covidshield \| covishield \| covid shield \| Oxford Vaccine \| Oxford vaccine \| Oxford vaccines \| Oxford vaccination \| OXFORD vaccine \| OxfordAZ vaccine \| oxford VACCINE \| Oxford Coronavirus Vaccine \| Oxford Coronavirus vaccine \| Oxford coronavirus vaccine \| Oxford COVID vaccine \| Oxford COVID-19 vaccine \| Oxford COVID shot \| Oxford Covid-19 vaccine \| Oxford University COVID-19 vaccine \| Oxford University vaccine \| Oxford Covid vaccine \| Oxford covid vaccine \| OxfordVaccine \| oxfordvaccine \| Oxford jab \| Oxford vax \| Oxford-AZ \| Oxford/AZ \| Oxford AZ |
| Moderna | MODERNA \| Moderna \| Modernas \| Modera \| ModeRNA \| Maderna vaccine \| Maderna \| moderna \| maderna \| SpikeVax \| Spikevax |
| Pfizer | Pfizer \| pfizer \| PFIZER \| Pfyzer \| Pfeizer \| Pfizers \| Pfiser \| PFizer \| Pfitzer \| pfiser \| Pfizers vaccine \| Phizer shots \| phizer shot \| Pfizer jabbed \| Pfiezer \| Pfiizer \| PHIZER \| Pfzier \| Pfzer \| pfeizer \| Pzizer \| pzifer \| Pizer \| Pzifer \| Pifzer \| fizer \| Phizener \| Phizer \| phizer \| P-Brand Vaccine \| BioNTech \| Biontech \| BioNtech \| biotech \| BioNTTech \| BNTX vaccine \| BNT162b2 \| BNT162 \| BionTech \| BNTX \| biontech \| Pz/B Tec vaccine \| PFE vaccine \| PPE vaccines \| PFZ vaccine \| PFE.N \| PFE \| pfe \| COMIRNATY \| Comirnaty |
| JNJ | JohnsonAndJohnson \| JohnsonandJohnson \| Johnsonandjohnson \| Johnson and Johnson \| Johnson &amp; Johnson \| Johnson&amp;Johnson \| Johnson Johnson \| JohnsonJohnson \| JOHNSON &amp; JOHNSON \| JOHNSON JOHNSON \| Johnson Johnson coronavirus vaccine \| Johnson Johnson's vaccine \| Johnson Johnson vaccines \| Johnson Johnson shot \| Johnson and johnson \| Johnson Johnson Covid-19 vaccine \| Johnson Johnson Covid vaccine \| Johnson Johnson COVID vaccine \| johnson and johnson \| johnson and johnson vaccine \| johnsonandjohnson \| johnson And Johnson \| johnson johnson \| JOHNSON JOHNSON COVID-19 VACCINE \| JOHNSON AND JOHNSON \| Johnson for vaccine \| Johnson COVID-19 Vaccine \| Johnson COVID-19 vaccine \| Johnson's COVID-19 Vaccine \| Johnson's Covid-19 vaccine \| Johnson's one-shot \| Janssen COVID-19 Vaccine \| Janssen COVID-19 vaccine \| JOHNSON COVID-19 VACCINE \| Johnson vaccine \| Janssen vaccine \| Janssen \| janssen \| JANSSEN \| Janssen/J J \| JNJ COVID-19 Vaccine \| JNJ COVID-19 VACCINE \| JNJ COVID-19 vaccine \| JNJ Covid vaccine \| JNJ Covid-19 vaccine \| JNJ Vaccine \| JNJ covid vaccine \| JNJ shot \| JNJ single-dose vaccine \| JNJ vaccine \| JNJ vaccine shot \| JNJ vaccine single shot \| JNJ vax \| JnJ vaccine \| jnj vaccine \| J J/Janssen COVID-19 vaccine \| J J Covid-19 Vaccine \| J J Covid-19 vaccine \| J J COVID - 19 VACCINE \| J J COVID-19 vaccine \| J J COVID vaccine \| J J covid vaccine \| J J Vax \| J J jab \| J J vac \| J J Vaccine \| J J vaccine \| J J vaccines \| J J VACCINE \| J J vaccination \| J J vaccinated \| J J vaccinations \| J J vaxx \| J J vaxxed \| J J shot \| J J shots \| J J one shot \| J J Covid shot \| J J covid shot \| J J Covid vaccine \| J J’s vaccine \| J J's vaccine \| J J/Janssen vaccine \| JJ vax \| J and J shot \| J and J \| J and J vaccine \| J J vax \| J/J \| j j shot \| J J/Janssen \| j j vax \| j j vaccine \| j and j \| J&amp;J \| JandJ \| J &amp; J vaccine \| JJ shots \| JJ shot \| JJ vaccine \| J vaccine |

Table S6. Keywords for the selection of users who self-reported positive. Users who post word combinations from the "Verb" and "Noun" columns or keywords from the "Other" column on Twitter are identified as infected.

| **Verb** | **Noun** | **Other** |
| --- | --- | --- |
| get \| got \| have \| had \| diagnose \| diagnosed \| diagnose with \| diagnosed with \| catch \| caught by \| infected by | covid \| corona \| ncov \| covid-19 \| covid19 \| coronavirus \| koronavirus \| sars-cov-2 \| covd \| virus \| a virus \| the virus | was positive \| were positive \| test positive \| tested positive \| identified by test \| recognized by test |
